# The oxytocinergic system in PTSD following traumatic childbirth: endogenous and exogenous oxytocin in the peripartum period

**DOI:** 10.1007/s00737-019-00994-0

**Published:** 2019-08-06

**Authors:** A. B. Witteveen, C. A. I. Stramrood, J. Henrichs, J. C. Flanagan, M. G. van Pampus, M. Olff

**Affiliations:** 1 Department of Midwifery Science/AVAG, Amsterdam Public Health research institute, Amsterdam UMC, location VUmc, Van der Boechorststraat 7, P.O. Box 7057, 1007 MB Amsterdam, The Netherlands; 2 Department of Obstetrics and Gynaecology, Amsterdam UMC, location AMC, Meibergdreef 9, Amsterdam, 1105 AZ The Netherlands; 3 Department of Psychiatry and Behavioral Sciences, Medical University of South Carolina, 67 President St., Charleston, 29425 SC USA; 4 Department of Obstetrics and Gynaecology, OLVG, Oosterpark 9, Amsterdam, 1091 AC The Netherlands; 5 Department of Psychiatry, Amsterdam UMC, location AMC, Meibergdreef 9, Amsterdam, 1105 AZ The Netherlands; 6 Arq Psychotrauma Expert Group, Nienoord 5, Diemen, 1112 XE The Netherlands

**Keywords:** PTSD, Trauma, Childbirth, Functional neuroanatomical, Psychological, Oxytocin

## Abstract

Birth experiences can be traumatic and may give rise to PTSD following childbirth (PTSD-FC). Peripartum neurobiological alterations in the oxytocinergic system are highly relevant for postpartum maternal behavioral and affective adaptions like bonding and lactation but are also implicated in the response to traumatic events. Animal models demonstrated that peripartum stress impairs beneficial maternal postpartum behavior. Early postpartum activation of the oxytocinergic system may, however, reverse these effects and thereby prevent adverse long-term consequences for both mother and infant. In this narrative review, we discuss the impact of trauma and PTSD-FC on normal endogenous oxytocinergic system fluctuations in the peripartum period. We also specifically focus on the potential of exogenous oxytocin (OT) to prevent and treat PTSD-FC. No trials of exogenous OT after traumatic childbirth and PTSD-FC were available. Evidence from non-obstetric PTSD samples and from postpartum healthy or depressed samples implies restorative functional neuroanatomic and psychological effects of exogenous OT such as improved PTSD symptoms and better mother-to-infant bonding, decreased limbic activation, and restored responsiveness in dopaminergic reward regions. Adverse effects of intranasal OT on mood and the increased fear processing and reduced top-down control over amygdala activation in women with acute trauma exposure or postpartum depression, however, warrant cautionary use of intranasal OT. Observational and experimental studies into the role of the endogenous and exogenous oxytocinergic system in PTSD-FC are needed and should explore individual and situational circumstances, including level of acute distress, intrapartum exogenous OT exposure, or history of childhood trauma.

## Introduction

The observation that women may develop posttraumatic stress disorder following childbirth (PTSD-FC; DSM-5 American Psychiatric Association 2013) emerged only two decades ago (Ballard et al. 1995) and has gained increasing attention ever since (Grekin and O’Hara 2014; National Institute for Clinical Excellence 2014). PTSD-FC may develop when mothers or their partners experienced or witnessed an actual or threatened death or serious injury of the newborn or mother (American Psychiatric Association 2013). PTSD is characterized by persistent symptoms of re-experiencing traumatic events, avoidance of distressing trauma-related stimuli, negative alterations in cognitions and mood, and hyperarousal (American Psychiatric Association 2013). Prevalence rates of traumatic childbirth vary from 9 to 20% depending on the exact wording of the A criterion of PTSD (according to DSM-IV or DSM 5) (Ayers et al. 2009; Stramrood et al. 2012; Boorman et al. 2014). In community samples 3–4% and in high risk samples 16–19% of women (i.e., with maternal psychiatric history and/or obstetric morbidity) develop PTSD-FC (Grekin and O’Hara 2014; Yildiz et al. 2016). The risk of PTSD-FC increases when peripartum vulnerability factors, such as fear of childbirth, severe health complications, operative delivery, and comorbid depression, are present (Grekin and O’Hara 2014; Koen et al. 2016; König et al. 2016; Ayers et al. 2016). PTSD-FC has a negative impact on family relationships, parent-to-infant bonding, future family planning, and subsequent infant development (McDonald et al. 2011; Parfitt and Ayers 2014; Cook et al. 2018).

Rigorous research on interventions to prevent PTSD-FC is however lacking, despite its clear need (McKenzie-McHarg et al. 2015). Although randomized clinical trials (RCTs) have been undertaken to evaluate whether early interventions in women after (traumatic) childbirth prevent the development of PTSD-FC and/or its severity, the majority of trials in unselected postpartum women have been found ineffective (de Graaff et al. 2018). Results from early interventions are more promising in high-risk women such those having experienced emergency caesarian section (Horsch et al. 2017). Furthermore, evidence-based treatments for PTSD, cognitive-behavioral therapy (CBT), and eye movement desensitization reprocessing (EMDR) therapy (National Institute for Clinical Excellence, 2014) have scarcely been evaluated in women with PTSD-FC. A few case studies suggest potential effectiveness of CBT and EMDR to treat PTSD-FC (Ayers et al. 2007; Sandström et al. 2008; Stramrood et al. 2012) and a recent RCT showed that CBT compared with a wait-list control group significantly reduced PTSD-FC symptoms although to a similar extent as the wait-list group (Nieminen et al. 2017). This is not surprising since in the general population already up to one-third of PTSD patients does not benefit from these evidence-based psychological treatments (Bradley et al. 2005; Schottenbauer et al. 2008; Cloitre 2009) and specific preventive or curative interventions may be needed for these target groups (Baas et al. 2017).

Interestingly, add-on medications or hormones have been implicated to enhance treatment response in PTSD in non-obstetric populations (Dunlop et al. 2012). Synthetic hormones like cortisol and oxytocin involved in animal and human stress responses have gained attention in prevention and treatment of PTSD (Koch et al. 2014; Sijbrandij et al. 2015; van Zuiden et al. 2017; Garcia and Delahanty 2017; Birur et al. 2017) and in mothers with postpartum depression and attachment difficulties (Riem et al. 2012; Bakermans-Kranenburg and van IJzendoorn 2013; Kim et al. 2014). Oxytocin may potentially impact the aberrant activity in a neurocircuitry involved in emotional and cognitive processing found in non-obstetric PTSD samples (Sherin and Nemeroff 2011; Zoladz and Diamond 2013; Koch et al. 2014) such as hyperresponsiveness of the amygdala to fearful stimuli reflected in heightened arousal and vigilance seen in PTSD (Shin et al. 2005; Protopopescu et al. 2005). Furthermore, decreased activity of the medial prefrontal cortex (PFC) and diminished structural and functional connectivity between the PFC and amygdala reflect inadequate top-down control over the hyperresponsive amygdala, clarifying the lack of suppression of attention to trauma-related stimuli and the diminished fear—inhibition and extinction in PTSD (Liberzon and Sripada 2008; Zoladz and Diamond 2013; Koch et al. 2014). This lack of top-down control of the mPFC over the amygdala might however be reversed through successful psychotherapeutic PTSD treatment with (adjunct) medications or hormones (Quidé et al. 2012).

Around birth, neuroendocrine interactions between the oxytocinergic system, the hypothalamus-pituitary-adrenal (HPA) axis, and the dopaminergic system are highly relevant for lactation and bonding, for protection against potential threats, for buffering the stress response, and for the motivation to raise offspring (Love 2014; MacDonald and Feifel 2014; Cox et al. 2015). Findings from studies in rats indicate that maternal postpartum behavior negatively affected by peripartum stress can be successfully targeted by early postpartum activation of the oxytocinergic system (with an oxytocin receptor agonist) thereby limiting the adverse life-long consequences of perinatal stress for both infant and mother (Maccari et al. 2014; Gatta et al. 2018). Because the oxytocinergic system is implicated in both PTSD and the peripartum period, we set out a narrative review to discuss the theoretical and empirical basis for the impact of oxytocin on women traumatized following childbirth. Findings from salient research papers on the therapeutic potential of exogenous oxytocin will be reviewed thereby highlighting areas for further research in clinical and neurobiological aspects of PTSD-FC.

## The oxytocinergic system in the peripartum period

**Fig. 1:**
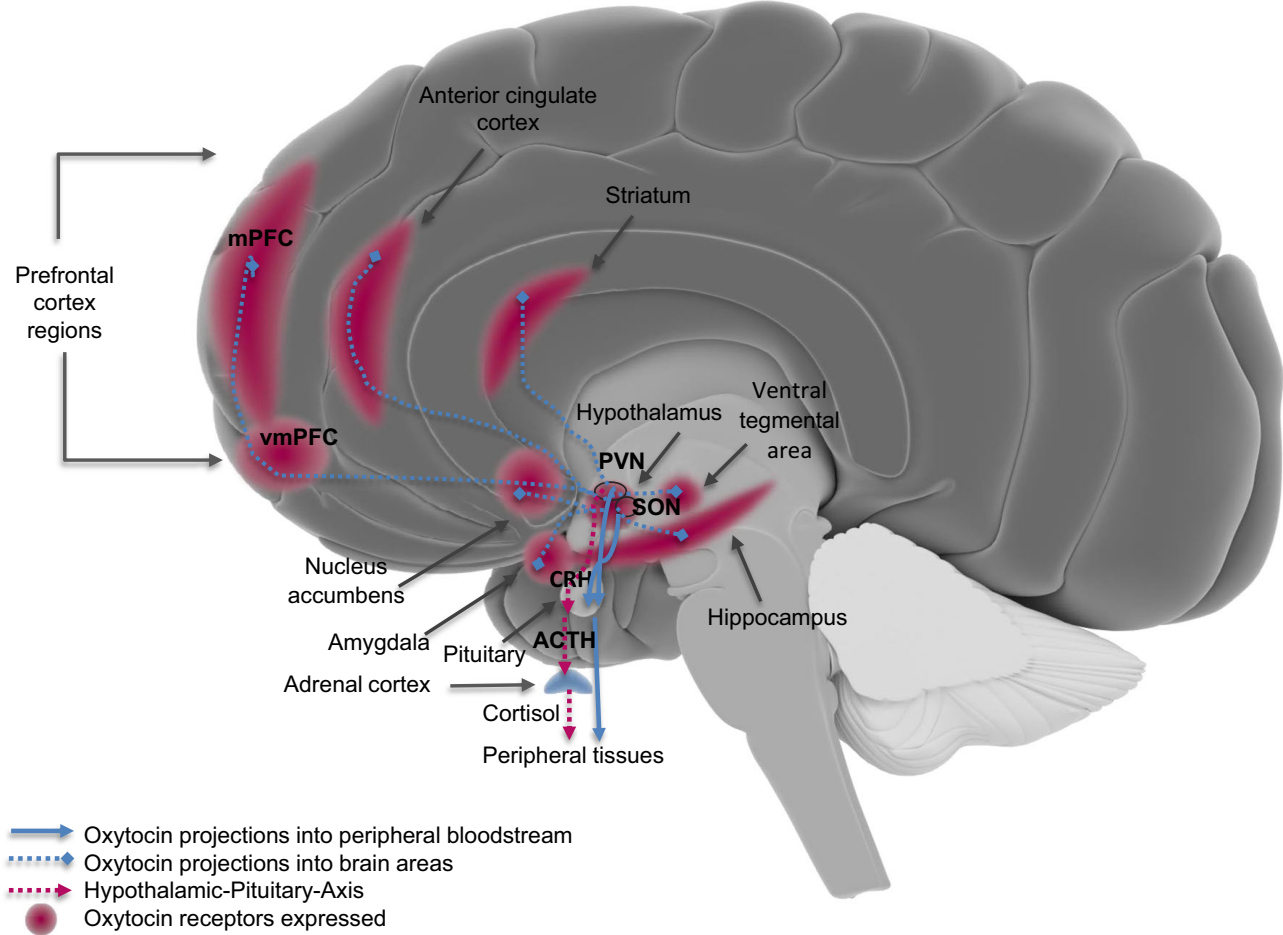
Simplified schematic representation of oxytocinergic projections to mid- and frontal brain areas and the hypothalamic-pituitary axis (based on animal and human models). PVN, paraventricular nucleus; SON, supraoptic nucleus; CRH, corticotropin-releasing-hormone; ACTH, adreno-cortico-tropic-hormone; (v)mPFC, (ventro)medial prefrontal cortex

The neuropeptide oxytocin (OT) is synthesized in the hypothalamic periventricular and supraoptic nuclei (PVN and SON) and projected to the pituitary gland from where it is released into the peripheral bloodstream (Fig. 1). OT is also centrally released from hypothalamic neurons that directly project into cerebral mid- and frontal regions (such as (pre)frontal cortices and amygdala) (Meyer-Lindenberg et al. 2011; Acevedo-Rodriguez et al. 2015). Peripheral and central oxytocinergic actions are induced by OT binding to oxytocin receptors (OTRs) that are highly available in the uterus, particularly at the start of labor. For central actions, OTRs are highly available in limbic areas like the amygdala and hippocampus that are also implicated in the traumatic stress response and PTSD (Brunton et al. 2012; Bell et al. 2014) (Fig. 1). OT interacts with the HPA-axis system to buffer the stress response and with dopamine release from the mesolimbic (reward) system directing motivational behavior to approach and engage in social activities (Skuse and Gallagher 2009; Smith and Wang 2012). There are several findings that support these interactions, such as the synaptic contacts between oxytocin- and corticotropin-releasing hormone (CRH)-expressing neurons found in the PVN of rats (Dabrowska et al. 2011) and OTRs found in key areas of the mesocorticolimbic system, such as the ventral tegmental area and nucleus accumbens of rodents that influence motivated behavior (Love 2014).

Around childbirth, OT mRNA expression and OTRs in the hypothalamic nuclei are significantly increased as shown in studies of rodents (Hillerer et al. 2014). OT neurons in the hypothalamus that project to parts of the limbic system such as the amygdala that interconnects with the mPFC show increased dendritic branching and synaptic alterations indicating great plasticity of the maternal brain in this period (Hillerer et al. 2014; Kim and Strathearn 2016). Immediately after birth, hypothalamus-induced neuroendocrine alterations support maternal behavioral adaptation through interactions with the mesocorticolimbic dopamine system, important for motivating behavior and attention directed towards the offspring (Kim and Strathearn 2016). These OT interactions with the dopaminergic mesolimbic system determine and regulate the maternal “reward” gained from raising an offspring as shown in several experimental studies of rats and mice (Olazábal 2018). In humans, maternal peripheral OT levels and attachment quality have been found associated with activity in reward-related functional neuroanatomic areas, such as the striatum, insula, and nucleus accumbens (Strathearn et al. 2009).

Evidence from preclinical research in rats shows that activity of the OTRs within the medial PFC plays a role in modulation of maternal care and reduction in postpartum anxiety-like behavior and illustrates the relevance of this brain area in the postpartum period and potentially in PTSD-FC (Sabihi et al. 2014). Blockage of OTRs within the medial PFC of rats prevents the normal attenuation of maternal anxiety and increases maternal aggressive behavior (Sabihi et al. 2014). In human studies, higher ante- or postpartum endogenous OT levels have been found associated with positive maternal caregiving behavior, mother-infant bonding, and maternal attachment behavior, particularly in women with more psychosocial stress and anxiety (Strathearn et al. 2012; Zelkowitz et al. 2014; Samuel et al. 2015). However, the normal association between higher peripartum endogenous OT secretion and positive parenting was found disrupted in mothers with adverse childhood experiences (Julian et al. 2018). Furthermore, studies in rats have shown that acute or chronic psychological stress in the perinatal period may hinder the normal adaptive changes in neural plasticity such as the increase of OT mRNA expression and OTRs in the hypothalamus, resulting in a reduction of OTR bindings in relevant brain areas such as the amygdala (Hillerer et al. 2011; Kim and Strathearn 2016). Recently, dysfunctional interactions between the oxytocinergic and dopamine systems have been thought to be involved in mood and postpartum depression (Post and Leuner 2018).

## Endogenous and exogenous OT in trauma and PTSD

After trauma exposure, women generally show higher endogenous OT levels (Seng et al. 2013; Olff et al. 2013) while lower levels have been found in traumatized men (Cao et al. 2014; Frijling et al. 2015). Higher endogenous OT levels in women suggest a necessity to decrease stress reactivity and may indicate an adaptive biological mechanism that motivates social contact-seeking in order to reduce interpersonal distress and conflict (Taylor et al. 2000; Bartz et al. 2011). This is in line with higher OT levels found in trauma-exposed women that were associated with cooperativeness and seeking social support (Nishi et al. 2015). Peripheral OT levels can be increased by affiliation or by exogenous administration and this may have anxiolytic effects (Taylor et al. 2006, 2010). For example, positive OT-induced autonomous responses to social stimuli have been found in women (Ditzen et al. 2013), corresponding with the affiliative response to social stressors resulting in higher endogenous OT levels in women (Taylor et al. 2000). However, in contrast to healthy men who show decreased amygdala activation after intranasal OT administration (Kirsch et al. 2005; Domes et al. 2007), healthy women show increased amygdala activation in reaction to threat-related or emotion-arousing stimuli after OT administration (Domes et al. 2010; Lischke et al. 2012; Rilling et al. 2014; Feng et al. 2015).

Table 1 provides an overview of neuroanatomic and psychological findings from RCTs published in peer-reviewed journals of intranasal OT administration in trauma-exposed female or mixed samples with or without PTSD. Overall, findings indicate beneficial psycho-physiological effects in samples of female or mixed PTSD patients such as restored sympathetic cardiac tone and a trend of reduced symptoms of PTSD (e.g., avoidance) and depression when added to prolonged exposure (Sack et al. 2017; Flanagan et al. 2018b), increased compassion towards women (Palgi et al. 2016), and improved anhedonic symptoms (i.e., motivation to engage in activities). OT restored neural responsiveness in regions of the reward pathway in trauma-exposed police officers with and without PTSD and the effect of OT on striatal responsiveness was strongest in individuals with higher anhedonia levels (Nawijn et al. 2016). OT administration also significantly improved aberrant functional neural responses to social rewards in mixed PTSD samples, up to the level found in healthy trauma-exposed individuals in the placebo condition (Nawijn et al. 2017). Interestingly, in men but not women with PTSD, OT increased recognition of body motions of anger (Palgi et al. 2017). This is in line with notion that OT alters the processing of social stimuli such as the salience of interpersonal cues but that behavioral effects depend on the individual and situational aspects (Bartz et al. 2011). For example, OT effects on behavioral performance were modulated by individual differences in sociability with improved performance in women scoring low but decreased performance in women scoring high on agreeableness (Groppe et al. 2013).

**Table 1:** OT administration trials in trauma-exposed individuals and PTSD patients

Year	Authors	RCT	Sample	Sex (f)	Dose (IU)	Min. to task	Stimuli/task	Neuroanatomic, psychophysiological and neuroendocrine response to OT (vs PL)	Psychological responses to OT (vs PL)
2017	Sack et al.	DBPLWS	35 PTSD	100%	24	45	Trauma-script driven imagery	↑ HR at baseline and to trauma-script ↓ marker sympathetic cardiac control ≠ HRV (parasympathetic cardial tone)	↓ total state PTSD symptoms ↓ avoidance (trend for significance) ≠ re-experiencing or dissociation
2016	Palgi et al.	DBPLCO	32 PTSD 30 healthy controls	28%	24	45	Compassion task	-----	PTSD and HC: ↑ compassion towards women (not towards men)
2017	Palgi et al.						Emotional/cognitive empathy tasks	-----	PTSD males: ↑ recognition body-motions of anger
2016	Nawijn et al.	DBPLCO	35 PTSD 37 trauma-exposed	44%	40	50	Monetary incentive delay task	PTSD and TE: ↑ left and right STRIA and INS (PTM) and right dACC PTSD: ↑ left vSTRIA during reward anticipation related to severity of anhedonia	PTSD: ↓ anhedonia
2017	Nawijn et al.						Social incentive delay task	PTSD and TE: ↑ left aINS and right PTM PTSD: ↑↑ left aINS (up to level of TE controls) PTSD symptoms ≠ PTM or INS responses PTSD and TE: ≠ AMY	PTSD and TE: ≠ social reward/punishment ratings and social reward feedback; PTSD: ↓ punishment ratings related to severity of PTSD symptoms
2016a	Koch et al.	DBPLCO	37 PTSD 40 trauma-exposed	47%	40	45	Fearful-angry/ happy-neutral faces	TE: ↑ left AMY PTSD: ↓ left AMY (independent of sex and stimuli valence)	PTSD: ↓↓ AMY related to higher state anxiety
2016b	Koch et al.						Resting-state	PTSD males:↑ FC vmPFC and CeM PTSD females: ↓ FC AMY-dACC	↓ anxiety and nervousness in PTSD
2018b	Flanagan et al.	DBPLBS	6 PTSD –OT 7 PTSD - PL	18%	40	45	PE session (weeks 2–9)	-----	↓ lower PTSD and depression symptom severity (non sign)
2016a	Frijling et al.	DBPLBS	19 TE-OT 18 TE - PL	51%	40	45 55–88	Script-driven imagery Resting-state	↓ FC AMY-left vlPFC ↑ FC AMY–INS ↓ FC AMY–vmPFC	↑ higher flashback intensity ↓ sleepiness during trauma-script
2016b	Frijling et al.	DBPLBS	23 TE-OT 18 TE - PL	59%	40	45	Emotional face-matching task	↑ right AMY to fearful faces ↑ left AMY in females to neutral faces	OT and PL: acute PTSD symptomatology ≠ with AMY responses to fearful faces
2017	van Zuiden et al.	DBPLBS	53 TE-OT 54 TE-PL	50%	40 (twice daily - 8 days)	12 days after trauma	-----	-----	≠ PTSD symptoms 1,5 months postpartum ↓ PTSD symptoms at follow up when high initial acute PTSD symptoms

↑, increased; ↓, decreased; ≠, no effect/no difference/unrelated; ----, not measured; *CO*, cross over; *WS*, within subjects; *BS*, between subjects; *DB*, double-blind; *PL*, placebo; *HR*, heart rate; *FC*, functional connectivity; *OT*, oxytocin, *d*, dorsal, *a*, anterior; *v*, ventral; *vl*, ventrolateral; *vm*, ventromedial; *AMY*, amygdala; *PFC*, prefrontal cortex; *ACC*, anterior cingulate cortex; *INS*, insula; *STRIA*, striatum

An OT-related reduction in amygdala activation in response to social stimuli was found in a mixed sample with PTSD compared with trauma-exposed controls which instead showed enhanced amygdala reactivity to these stimuli after OT (Koch et al. 2016a). OT-related improved connectivity between ventromedial PFC and centromedial amygdala was found in male police officers with PTSD compared with controls (Koch et al. 2016b) while in women with PTSD, connectivity between two areas within a neural circuitry involving parts of the salience network (i.e., amygdala and dorsal anterior cingulate cortex) was increased in the placebo condition but diminished after OT administration (Koch et al. 2016b). The latter suggests anxiolytic effects of OT in this fear circuitry involved in processing of emotions in females.

Taken together, the aforementioned pattern of OT-related reduction PTSD symptoms and improvements of anhedonia and compassion reflected in an increase of reward-related functional activation and reduced amygdala activation indicates enhanced short-term sensitivity for social interactions that are of relevance for involvement and participation in emotionally demanding exposure therapy for PTSD. Recently, these beneficial effects of OT were also found in individuals with PTSD treated with OT (versus placebo) compared with trauma-exposed controls, as shown by improved working memory scores and enhanced functional connectivity between the dorsolateral PFC and anterior cingulate (Flanagan et al. 2018c).

More negative functional neuroanatomic and psychological effects of intranasal OT were however found in trials among individuals who were recently exposed to trauma such as traffic accidents. Increased fear processing during resting state (Frijling et al. 2016a) and amygdala activation to fearful and neutral faces in females (Frijling et al. 2016b) were found after OT administration in recently exposed trauma victims, in line with OT effects found in healthy females (Domes et al. 2010; Lischke et al. 2012). Individuals with recent trauma exposure also showed OT-related reduced functional connectivity between the PFC and amygdala, reflecting decreased top-down control over the hyperactive amygdala and increased re-experiencing intensity (Frijling et al. 2016a, b). Repeated OT administration for 8 consecutive days starting maximum 12 days after trauma exposure also showed no difference in PTSD symptom severity at 1.5 months after trauma exposure compared with placebo (van Zuiden et al. 2017). However, trauma-exposed individuals with a high level of initial acute distress reported lower PTSD symptoms at follow-up after having received OT repeatedly compared with individuals with initial high distress in placebo condition (van Zuiden et al. 2017), indicating potential of intranasal OT exclusively for those with high acute distress levels after trauma.

## Endogenous and exogenous OT in the peripartum period

Throughout pregnancy, the majority of women show a slight increase of endogenous OT levels and a subsequent decline at 2 months postpartum (Feldman et al. 2007; Levine et al. 2007; Prevost et al., 2014; van der Post et al. 1997). During the early postpartum (lactation) period, endogenous OT levels are generally elevated due to increased cerebral OT in order to buffer stress reactivity (e.g., Bell et al. 2014; Cox et al. 2015; Grewen et al. 2010) with reduced perception of negative environmental stimuli (Altemus et al. 2001; Heinrichs et al. 2001). Intravenous OT administration for augmentation of labor is a frequently performed obstetric intervention (i.e., 25%) in most Western countries (Boie et al. 2018). However, it has been postulated that this might interfere with normal neurobiological adaptations around birth, thereby potentially affecting maternal and infant mental health. Despite some positive associations of intrapartum synthetic OT with higher plasma OT levels postpartum (Gu et al. 2016), lower aggression (Prevost et al. 2014), and higher socialization during breastfeeding (Jonas et al. 2009), other studies point to adverse outcomes associated with high doses of intrapartum synthetic OT such as less successful breastfeeding (Olza Fernández et al. 2012; Gu et al. 2016) and negative emotional wellbeing of the mother postpartum (i.e., depression and anxiety but not PTSD-symptoms) (Gu et al. 2016). Findings from a large retrospective population-based study also suggest that women exposed to intrapartum synthetic OT have a greater risk of developing depressive and/or anxiety disorders within the first year postpartum as compared with women not exposed to intrapartum synthetic OT, irrespective of pre-pregnancy depression or anxiety or mode of delivery (Kroll-Desrosiers et al. 2017). These potential adverse effects of synthetic intrapartum OT could be explained by desensitization of the OTR due to excessive amounts of synthetic OT. More specifically, due to OT agonist stimulation, a (temporarily) decrease in binding sites takes place (Conti et al. 2008) and, based studies in rats and mice, Gimpl and Fahrenholz suggest that internalization of the human OTR may make OTRs unavailable for further OT binding (Gimpl and Fahrenholz 2001; Wahl 2004). However, the current state of scientific evidence does not allow us to make causal inferences regarding the association between the retrospectively assessed relationship of intrapartum synthetic OT with adverse wellbeing from nationwide collected health data.

In Table 2, results from OT administration trials in postpartum women are presented. After OT administration, an increase of reward-related mesolimbic functional activation in ventral tegmental area (but not nucleus accumbens) to images of crying infants in both postpartum and nulliparous women was found (compared with placebo) (Gregory et al. 2015). Findings are in line with preclinical studies showing increased maternal caretaking behavior after OT injections in the ventral tegmental area of rats (Pedersen et al. 1994), which suggests improved motivation for caretaking behavior under OT in healthy postpartum women. This is of importance for women with avoidant attachment styles who are at increased risk for developing PTSD-FC after operative deliveries (Ayers et al. 2014). However, activation of the amygdala (part of the salience network) was reduced in response to negative images after OT administration in nulliparous women, while a blunted amygdala reaction and reduced arousal to all types of infant images was found in postpartum women only in the placebo condition (Rupp et al. 2013, 2014). Again, saturation of available OTRs caused by already higher endogenous OT levels around birth could explain this blunted reaction to OT in postpartum women (Rupp et al. 2014).

**Table 2:** OT administration trials in postpartum women

Year	Author	RCT	Sample	Dose (IU)	Min. to task	Stimuli/task	Neuroanatomic, psychophysiological and neuroendocrine response to OT (vs PL)	Psychological responses to OT (vs PL)
2015	Gregory et al.	DBPLBS	29 breastfeeding mothers and 30 NP controls	24	30	IAPS images (sexual, neutral, or infant)	PP and NP: ↑ VTA (to infant and sexual images); ↔ NAc (to any image)	----
2013	Rupp et al.					IAPS images (sexual, neutral, crying infant, smiling infant)	PP (vs NP) under PL: ↓ right AMY activation to all images; PP (vs NP) under OT: ↔ right AMY to all images	PP (vs NP) under PL *and* OT: ↓ sexual arousal scores. ↓ arousal to infant images NP (vs PP) under OT: ↔ arousal to infant stimuli.
2014	Rupp et al.					IAPS images (neutral and negative)	PP and NP: ↔ cortisol PP (vs NP) under PL: ↓ rAMY to negative images NP (vs PP) under OT ↓ rAMY to negative images	PP (vs NP) under PL: ↓ arousal to negative images; PP (vs NP) under OT: ↔ arousal to negative images
2013	Mah et al.	DBPLWS	25 mothers with PPD (infants 3–12 months)	24	45	Infant interaction session	----	↓ mood ↑ ratings of their child as ‘difficult’ ↑quality of the mother-child relationship
2015				24	55	Enthusiastic stranger paradigm	----	↑ maternal protectiveness of their child ↓gaze duration
2017				24	30–55	Crying paradigm	----	↑ perceiving infant cry as urgent ↑ harsh caregiving strategy upon infant cry ↔ maternal sensitivity
2015	Clarici et al.	DBPLBS	5 mothers with PPD - OT and 11 with PPD – PL	16 daily	----	12 weekly psychodynamic therapy sessions	----	↔ depressive symptoms ↔ basic emotional traits (including attachment) ↓ self-centered in depressive presentation (therapist ratings)

↑, increased; ↓, decreased; ↔, no effect/similar; ----, not applicable/unknown; *CO*, cross over; *BS*, between subjects; *WS*, within subjects; *DB*, double-blind; *PP*, postpartum; *PPD*, postpartum depression; *NP*, nulliparous; *HC*, healthy controls; *PL*, placebo; *OT*, oxytocin; *VTA*, ventral tegmental area; *NAc*, nucleus accumbens; *AMY*, amygdala

In depressed postpartum women, intranasal OT (compared with placebo) in advance of infant interaction sessions, negatively affected self-reported maternal mood and child behavior although quality of mother-infant interaction improved (Mah et al. 2013). OT administration in postpartum depressed women also increased the salience of infant crying and maternal protectiveness (Mah et al. 2015, 2017). Maternal protectiveness is however an adaptive maternal postpartum reaction not linked to mood (Mayes and Leckman 2007). Nonetheless, OT also enhanced the potential of mothers to choose harsh caregiving strategies in response to crying infants (Mah et al. 2017). This is of importance, since mothers with depression already have an increased likelihood to be less emotionally responsive and to respond with more aggressiveness to their children and exogenous OT may thus increase this behavior (Drury et al. 2016). In healthy women, intranasal OT is associated with increased salience of threat-related stimuli (Domes et al. 2010) and aggressiveness, particularly to out-group members while OT tends to increase trustfulness to in-group members (De Dreu et al. 2010). In distressed women, OT may exert different effects than in healthy women, for example, poorer couple conflict behaviors were found in distressed substance abusing women after OT (Flanagan et al. 2018a). This, in sum, suggests that the potential effects of OT depend on women’s mood and circumstances (Bartz et al. 2011; Olff et al. 2013). Recent findings suggest that OT may not operate as expected in individuals with high levels of early (child) adversity and that the individuals’ social context should be carefully considered in the administration of OT (Graustella and Macleod 2012). Furthermore, intranasal OT was more beneficial on mid- and long-term (1.5 to 6 months) PTSD symptom course after acute trauma exposure in women with hormonal contraception use (resembling high levels of estrogens and low levels of progesterone) (Engel et al. 2019). The latter finding is in line with previous links suggested between hormonal status and PTSD risk in women (Garcia et al. 2018).

No additional effects of daily OT during the treatment period of 12 weeks on depression or on attachment-like emotional traits in postpartum depressed mothers were found in a small trial of Clarici et al. (2015). Only self-centeredness during treatment was significantly lower in the OT group compared with placebo (Clarici et al. 2015). This may reflect an OT-related increase of the motivation to socially interact which could be meaningful for the formation of a positive therapeutic alliance. Indeed, a positive social bond (such as trusted therapist) may be necessary for OT to exert its modulating effect (i.e., decrease of fear (and amygdala) in PTSD (Charuvastra and Cloitre 2008). As described earlier, OT administration in PTSD patients has shown to improve the motivation to engage in activities and compassion and to reduce nervousness and anxiety both reflected in an increase of reward-related functional activation and reduced activity and connectivity in the salience network (Koch et al. 2016a, b).

## Clinical implications and suggestions for future research

At the time of drafting this review, no trials of intranasal OT administered in postpartum (traumatized) women with (symptoms of) PTSD-FC were available. This indicates a critical unexplored area of research. The overview of literature presented in this review, however, provides some specific directives and refinements for future research in women with PTSD-FC and/or acutely traumatized women after childbirth. Research is needed to examine whether and how OT administration might effectively reduce or prevent PTSD-FC particularly in women at high-risk for PTSD-FC. Future studies should take into account that the effects of OT may be modulated by timing and dosage of OT administration. PTSD models in rodents have shown timing effects of OT administration in terms of traumatic memory consolidation and fear extinction (Toth et al. 2012; Eskandarian et al. 2013) and anxiolytic effects produced by repeated OT administration (Janezic et al. 2016).

Furthermore, findings reviewed in this article are predominantly based on immediate effects of short-term OT administration but the few studies available on effects of repeated intranasal OT administration point towards clinically meaningful symptom improvements in PTSD (Flanagan et al. 2018b) and acutely distressed individuals after trauma exposure (van Zuiden et al. 2017). Therefore, adequately powered trials are warranted to examine whether repeated low-dose intranasal OT administration is an early preventive intervention for PTSD-FC in women with a high level of acute childbirth-related distress. Future trials are also needed to evaluate whether repeated intranasal OT administration as a therapeutic adjunct has the ability to increase tolerability of prolonged exposure, enhance therapeutic alliance, and help with memory consolidation and fear extinction in PTSD in general and/or following childbirth (Clarici et al. 2015; Nawijn et al. 2016; Sack et al. 2017; Flanagan et al. 2018b). Furthermore, differential effects of postpartum intranasal OT in women already exposed to various amounts of intrapartum synthetic OT should be explored in future research.

The findings reviewed here also suggest differential effects of intranasal OT according to specific individual characteristics or circumstances of postpartum women (Bartz et al. 2011). For example, the effect of intranasal OT in women with PTSD-FC might be modified by comorbid depression (Kim et al. 2014), lactation (i.e., breastfeeding or formula-feeding) (Cox et al. 2015), history of early adversities (Pierrehumbert et al. 2010; Fan et al. 2015), or lack of social support or relationship distress (Taylor et al. 2006). In PTSD-FC, this picture is further complicated by normal neuroendocrine and functional neuroanatomic alterations around parturition (e.g., reduced stress responsiveness in the HPA axis and blunted reaction to intranasal OT). Therefore, future research should also examine the endogenous OT system in the development and maintenance of PTSD-FC.

### Conclusions

Collectively, the evidence from non-obstetric traumatized samples with or without PTSD and from peripartum healthy or depressed samples implies an important role for the oxytocinergic system in the neuroendocrine dysregulations in women with (or at high risk for) PTSD-FC. The restorative functional neuroanatomic and psychological effects of OT further strengthen the motivation to examine the ability of OT to augment psychotherapeutic interventions in PTSD-FC with or without comorbid depression. Despite availability of reviews on intranasal oxytocin in postpartum depression (Kim et al. 2014; Moura et al. 2016) and parenting behavior (van IJzendoorn and Bakermans-Kranenburg 2016; Szymanska et al. 2017), fundamental observational research is necessary to delineate the nuances of the oxytocinergic system in PTSD-FC. Moreover, the potentially adverse effects of intranasal OT on mood and the increased fear processing and reduced top-down control over amygdala activation in women with acute trauma exposure warrant cautionary use of intranasal OT in postpartum women. For acutely traumatized postpartum women, preventive interventions that aim at social (i.e., partner) and obstetric staff support in order to further promote endogenous oxytocin release may be more beneficial in preventing PTSD-FC (van Heumen et al. 2018; van Steijn et al. 2019). Finally, because individual and situational circumstances matter, future clinical research trials that provide possibilities to examine the more individualized therapeutic response of exogenous OT, for example by using personalized methods, might give directions on who will obtain most benefit from exogenous OT.
